# The Potency of Invisalign® in Class II Malocclusion in Adults: A Narrative Review

**DOI:** 10.7759/cureus.49664

**Published:** 2023-11-29

**Authors:** Feras Y Dahhas, Eman M Al-saif, Albatool M Alqahtani, Nizar F Al Farraj, Maryam A Alshaikh, Bshaer S Almadhi, Nada Albuolayan, Haneen H Alhayaza, Nada A Asiri, Khulud H Alshaya

**Affiliations:** 1 Dentistry, Al-Noor Specialized Hospital, Jeddah, SAU; 2 Orthodontics, King Fahd University of Petroleum and Minerals, Qatif, SAU; 3 Dentistry, Ministry of Health, Khamis Mushait, SAU; 4 Dentistry, King Salman Armed Forces Hospital, Tabuk, SAU; 5 Dentistry, Taibah University, Jeddah, SAU; 6 General Dentistry, Badr Al Faraj Dental Clinic, Jeddah, SAU; 7 Dentistry, Presidency of State Security, Riyadh, SAU; 8 General Dentistry, Ministry of Health, Abha, SAU; 9 General Dentistry, Magrabi Dental Center, Jeddah, SAU; 10 General Dentistry, Aseer Central Hospital, Abha, SAU

**Keywords:** malocclusion, digital orthodontics, class ii malocclusion, invisalign, orthodontic management

## Abstract

In recent years, a greater number of adult patients are seeking orthodontic treatment, not only for esthetics but for better functioning and hygiene purposes. However, they are more focused on comfortable and invisible treatment alternatives to conventional metal brackets. This abstract is a multifaceted interplay between Invisalign^®^ and different treatments of class II malocclusion, which embarks on the potency of Invisalign^®^ in treating this condition in adult patients. The review delves into analyzing the efficacy of Invisalign^®^ in molar distalization, class II elastics, extraction treatment, class II division 2 patients, their limitations, challenges, and future prospects. This article aspires the orthodontists understand the complex nature of class II malocclusion treatment in adults with Invisalign^®^ and its application in clinical practice with improved patient outcomes.

## Introduction and background

Class II malocclusion is the prevailing issue encountered in orthodontic practices. The prevalence of this malocclusion is 37% in European countries and 33% in the United States [1]. However, in Saudi Arabia, the pooled prevalence of Angle's class II malocclusion is comparatively less (17.84%) [2].

Depending on the first molar relationship, Angle classified class II malocclusion as division 1 and division 2 [3]. Class II division 1 malocclusion is characterized by class II/end-on molar relationship along with proclination of maxillary incisors, whereas clinical manifestation of class II division 2 malocclusion is class II/end-on molar relationship with retroclined maxillary central incisors with overlapping lateral incisors, often associated with deep bite and minimal overjet. Class II malocclusion may also manifest as a subdivision where the molar relation is class I on one side and class II/end-on on another side [4]. Angle did not include skeletal factors while classifying class II malocclusion. Therefore, this condition was further classified as dental, skeletal, or functional [5]. The literature outlines various techniques for addressing class II malocclusion for different age groups. For growing patients, the treatment approach is usually growth modification depending on the jaws involved. In adult patients, dental class II division 1 malocclusions are usually treated by extracting either maxillary or both maxillary and mandibular premolars. If the underlying etiology for an established class II malocclusion is a severe skeletal discrepancy, then orthognathic surgery is the only treatment option.

Nowadays, because of social media platforms, there is increased awareness about orthodontic treatment, especially among adults. However, these patients are very much conscious about their social exposure and life, which makes them opt for clear aligners. Invisalign^®^ is the leading brand of clear aligners, which was developed by Zia Chisti, a Stanford University student. These aligners were registered under Align Technology, San Jose, California, in 1997 [6]. Invisalign^®^ is widely chosen by orthodontists and patients because of its comfort, superior esthetics, and authentic services. Currently, almost 7.5 million Invisalign^®^ cases have been dispatched worldwide. Their yearly net revenues exceed $2.3 billion [7]. Invisalign^®^ system is considered to be a viable alternative to conventional therapy for correcting mild to moderate malocclusions without the need for extraction, specially designed to control various tooth movements, which are usually not achievable by their predecessors. Studies that compared predicted versus achieved tooth movement showed the highest predictability with distalization of maxillary molars (88%) [8]. Recently, align technology has introduced an innovation known as "wings" within their aligner system that aims at rectifying class II malocclusion through mandibular protraction, which is the first US FDA-approved clear aligner treatment [9].

The aligner approach is entirely different when it comes to treating class II division 2 cases. A multivariate regression study was conducted to determine the effectiveness of Invisalign^®^ in proclination and intrusion of maxillary incisors, which were found to be 69.8% and 53.3%, respectively [10]. To correct the molar relationship, temporary anchorage devices (TADs) and different attachments were used. However, there are certain limitations to this system, which include challenges in vertical changes, overjet correction, buccolingual movements, occlusal relationships, and jaw expansion [11,12]. To overcome some of these limitations, align technology has developed a powerful tool known as SmartForce™. These attachments are like anchors that add grip and dexterity to this aligner system, providing orthodontists predictability and control over tooth movement. Several clinical factors influence the determination of the best force system for a particular tooth movement. These factors include the accurate determination of the center of resistance of each tooth, the application of low constant force for optimal biological response, and the consideration for the potentially slow physiological response of mature adults to orthodontic forces. Optimized attachments are strategically engaged by initial aligners in the series, allowing time for the patient’s physiological response and improving treatment tracking from the outset [13].

The treatment options with Invisalign^®^ in adult patients are broadly categorized mainly into correction of class II division 1 and division 2 malocclusion. This literature review tries to seek the potential pieces of evidence for treating class II malocclusion with Invisalign^®^ in adult patients and the possible outcomes of the treatment.

## Review

Research methodology

An electronic database search was performed to gather the articles on this topic. For this purpose, PubMed, Embase, MEDLINE, and Google Scholar databases were utilized employing the following keywords: "Invisalign, Clear Aligners, Invisalign + Class II, Clear Aligners + Class II, Class II + adults." The time period for published articles was between 1970 and 2023.

Inclusion Criteria

Articles in English; full-text research; prospective or retrospective studies including randomized controlled trials, case series, and case reports; review papers; and books were included in this study.

Exclusion Criteria

Studies in any language other than English, research with irrelevant outcomes or outcomes unrelated to this topic, journals that are not cited in the open access checklist for predatory publishers, and published articles before January 1, 1970, were excluded from this study.

Impact of Invisalign^®^ on adult patients with class II malocclusion

The effect of age on orthodontic tooth movement has been scientifically proven in animals. According to the evidence in the literature, the amount and velocity of tooth movement in older individuals are comparatively slower than their younger counterparts [14]. Invisalign^®^ is no option for this. The impact of Invisalign^®^ treatment on adult patients with class II malocclusion is significant, particularly in terms of reducing overjet (OVJ), improving the molar relationship, and repositioning the upper incisors while effectively controlling the inclination of both upper and lower incisors. Notably, as discussed in research by Rongo et al., the inclination of lower incisors is a critical factor in such treatments, with Invisalign's ability to prevent unwanted proclination in this context. This advantage becomes particularly crucial in cases where mandibular symphysis bone fragility poses a risk of periodontal issues. This study emphasizes the consistent and predictable results of Invisalign^®^ in terms of upper molar distalization and the maintenance of incisor inclination, attributes that are critical for achieving successful class II malocclusion correction in adult patients [15]. While acknowledging the existing limitations, the research points to the potential of Invisalign^®^ treatment in conjunction with class II elastics, highlighting its advantages over other treatment options that may pose anchorage issues or adverse effects on dentition. Nonetheless, the authors stress the need for further comprehensive studies to establish clear guidelines for the combined use of Invisalign^®^ and class II elastics, considering factors like patient compliance, occlusal adjustments, and the influence of specific dental conditions.

The potency of Invisalign^®^ in class II division 1 malocclusion in distalization of molars

A study by Saif et al. evaluated the efficiency of maxillary molar distalization using Invisalign^®^, and they found that Invisalign^®^ can effectively be used for adult patients needing maxillary molar distalization, particularly when a mean distalization movement of approximately 2.6 mm is required [16]. However, clinicians should be cautious of potential countereffects when planning distal movement of the maxillary molars, especially in cases where the patient initially presents with a significant overjet. To address this concern, it is advisable for clinicians to consider prescribing overcorrection or implementing the use of auxiliary measures at an earlier stage in the treatment process [17].

Contrary to this, research conducted by Patterson et al. aimed to determine the efficacy of clear aligners in treating class II malocclusion following the completion of initial aligner treatment. After a thorough examination of 80 patients under controlled conditions using techniques certified by the American Board of Orthodontics (ABO) Model Grading System, the study revealed that the impact of aligners was relatively modest. Specifically, the correction of anteroposterior issues in class II malocclusion patients was only 6.8% of the anticipated correction, while the overbite was corrected by 38.9% of the projected amount [18]. Consequently, the researchers concluded that the Invisalign^®^ system, in this context, failed to achieve substantial class II correction or reduce overjet significantly. Another study that supports this finding was recently documented. In this study, 32 adult patients with class II malocclusion who had undergone treatment with Invisalign^®^ by sequential distalization of maxillary molars were included. This study assessed the predictive value of ClinCheck^®^ Pro software with the final results and came across significant differences between the predicted and achieved values [19].

The potency of Invisalign^®^ in class II division 1 malocclusion with extraction

Some of the class II division 1 malocclusion patients require premolar extraction to correct the overjet and molar relationship. Several studies have been conducted to evaluate the efficacy of Invisalign^®^ in en masse retraction of incisors and prevent anchorage loss. These studies demonstrated unwanted tooth movements with incisors, canines, and molars. Also, the central incisor retraction and first molar anchorage control were partially achieved [20,21]. These limitations of aligners in extraction space closure could be due to the stress-breaking effect created by extraction space, which causes limited expression of labial force in the anterior region. The second reason could be torque loss while closing the space. Also, the labial force on the maxillary anterior is lacking since there is no molar distalization involved.

The potency of Invisalign^®^ in class II division 2 malocclusion

There are usually four types of critical orthodontic tooth movements involved in correcting class II division 2 malocclusion, i.e., controlled intrusion, proclination and labial movement of maxillary incisors, and correction of class II molar relationship. The class I molar relation is usually achieved either by distalization of maxillary molars or by applying class II elastics, which mesializes lower molars. The patient-related factors that affect the proclination and intrusion of incisors include age, sex, tooth morphology, and root length [22]. On the other hand, the mechanical factors related to aligners are material, attachments, thickness of aligners, and production techniques [23].

A study evaluated the effectiveness of Invisalign^®^ in correcting class II division 2 in adult patients particularly pertaining to intrusion and proclination of maxillary incisors. In this study, pre- and post-treatment models were superimposed, and the difference between predicted and actual tooth movement (DPA) was analyzed. They found that achieved incisor proclination and intrusion were 69.8% and 53.3%, respectively [10].

A successful case of a 13-year-old boy with severe class II malocclusion division 2 was reported. In this case, Invisalign^®^ combined with class II elastics during the initial phase of treatment led to complete correction of the class II malocclusion, and the desired overbite and overjet were achieved. Notably, the overbite was reduced from 5 to 2 mm [24]. These case reports, along with others, suggest that clear aligners not only ensure proper oral hygiene and superior aesthetics but also hold the potential for reducing class II malocclusions when used in conjunction with appropriate treatment auxiliaries.

The potency of Invisalign^®^ with class II elastics

In class II malocclusion, when intermaxillary elastics are prescribed, the dentoalveolar effects that usually occur are lingual tipping of maxillary incisors along with some extrusion and mesial tipping of mandibular molars with extrusion and intrusion of lower incisors. This temporarily causes clockwise rotation of the occlusal plane that comes to its original form at the end of the treatment.

Elastics with attachment auxiliaries in Invisalign^®^ have been reported to be an effective way of correcting molar relationships in class II malocclusion. A case report of an adult patient with class 2 subdivision malocclusion was documented with a successful outcome of a corrected molar relationship [25]. Another study evaluated the effects of Invisalign^®^ and class II elastics in adult patients with class II malocclusion. The class II elastics were engaged from precision cuts on upper aligners to tubes bonded on lower molars. Clinically as well as statistically significant changes were found in overjet, retroposition of upper incisors, and molar relationship, which were -1.4 ± 0.2, -1.3 ± 1.7, and 0.75 ± 0.45, respectively. Regarding skeletal variables, no significant changes were observed [15].

The use of clear aligners demonstrates effective control over the extrusion and inclination of upper incisors, preventing excessive gum exposure and maintaining favorable smile aesthetics. Furthermore, the treatment with aligners offers a promising "bite effect," effectively stabilizing the mandibular plane and preventing molar extrusion often associated with class II elastics and fixed appliances.

Challenges and limitations of Invisalign^®^

In some cases, the Invisalign^®^ system may not be able to achieve all the necessary tooth movements required for comprehensive correction, particularly in complex cases involving skeletal discrepancies. As a result, additional refinements and alternative treatment modalities may be necessary to address specific issues that arise during treatment, such as posterior open bite incidence created after treatment with the initial set of aligners. In situations where significant class II correction or overjet reduction is required, the use of elastics in conjunction with Invisalign^®^ treatment may not consistently yield the desired outcomes, especially in the adult population. This emphasizes the need for a comprehensive assessment by an experienced orthodontist who can evaluate the severity of the malocclusion and determine the most suitable treatment approach [18].

Additionally, the incidence of posterior open bite during or after treatment suggests the need for careful monitoring and adjustments during the course of Invisalign^®^ treatment. Addressing such complications may require additional orthodontic interventions or adjustments to the treatment plan to ensure the desired outcomes are achieved. It is essential for both orthodontists and patients to have a clear understanding of the limitations and potential complications associated with using Invisalign^®^ for class II malocclusion. In complex cases, a combination of different orthodontic approaches, such as the use of other orthodontic appliances or even orthognathic surgery, may be necessary to achieve optimal results. Regular monitoring and communication between the orthodontist and the patient are crucial to address any issues that may arise during the treatment process and to ensure the best possible outcome for the patient's overall oral health and aesthetics.

Moreover, limitations arise when addressing specific dental conditions, such as premolar extractions, as the appliance struggles to maintain proper tooth positioning during space closure, leading to compromised outcomes [26,27]. Studies note challenges in the treatment of anterior open bites, with difficulties in achieving desired occlusion and potential positioning discrepancies between anterior and posterior teeth [28].

Considerations for orthodontists: best practices for Invisalign^®^ in class II malocclusion cases

When managing class II malocclusion cases using the Invisalign^®^ system, orthodontists must adopt a comprehensive approach that accounts for various clinical considerations and best practices. It is crucial to carefully select suitable cases for Invisalign^®^ treatment and accurately assess the severity of malocclusion, considering the limitations of the system in addressing complex skeletal discrepancies [1]. Educating patients about the significance of compliance and the necessity of wearing aligners for the prescribed duration each day is paramount for successful outcomes [29]. Creating a well-structured treatment plan that incorporates the use of attachments strategically placed for effective tooth movement control is crucial [13]. Regular monitoring and follow-up appointments are essential to assess the progress of treatment and make necessary adjustments to ensure desired outcomes are achieved [18]. Orthodontists should also be prepared to employ interdisciplinary collaboration and consider additional orthodontic interventions or surgical options for complex cases that may exceed the capabilities of the Invisalign^®^ system [30]. Through effective patient support and communication, orthodontists can guide patients through potential challenges, ensuring a positive treatment experience and improved compliance [15]. Continuous education and research are vital to stay updated with the latest advancements and refine treatment strategies for class II malocclusion cases using the Invisalign^®^ system [16].

## Conclusions

This narrative review, aimed at evaluating the efficacy of Invisalign^®^ in treating class II malocclusion in adults, has revealed nuanced findings specific to different treatment modalities. In terms of distalization, studies indicate that Invisalign^®^ is effective for adult patients requiring maxillary molar distalization, with a notable ability to achieve a mean distalization movement of approximately 2.6 mm. However, the system's limitations become apparent in cases with significant initial overjet, necessitating overcorrection or auxiliary measures. Regarding treatment involving extractions, the review found that while Invisalign^®^ can be employed in en masse retraction of incisors to prevent anchorage loss, it often leads to unwanted tooth movements and only partial achievement of desired molar and incisor positions. This suggests a reduced efficacy of Invisalign^®^ in managing space closure post-extraction, potentially due to stress distribution and torque loss challenges.

In cases involving class II elastics, Invisalign^®^ demonstrates a beneficial impact on molar relationships but with varying degrees of success. Some studies highlighted clinically significant changes in overjet reduction and molar relationships but also noted the absence of substantial skeletal changes. This underscores the importance of tailoring treatment plans to individual patient needs and suggests the potential need for additional orthodontic interventions in complex cases. In summary, while Invisalign^®^ presents as a versatile tool in the orthodontic arsenal, its application in class II malocclusion cases, especially in adults, requires careful case selection and a keen understanding of its capabilities and limitations. The treatment outcomes vary depending on the specific malocclusion characteristics and the chosen treatment approach, be it distalization, extraction, or the use of elastics. This review stresses the need for individualized treatment planning and suggests that while Invisalign^®^ is a valuable option in many cases, it may need to be complemented with other orthodontic methods for optimal results in complex malocclusions.
